# A Seed Preferential Heat Shock Transcription Factor from Wheat Provides Abiotic Stress Tolerance and Yield Enhancement in Transgenic *Arabidopsis* under Heat Stress Environment

**DOI:** 10.1371/journal.pone.0079577

**Published:** 2013-11-12

**Authors:** Harsh Chauhan, Neetika Khurana, Preeti Agarwal, Jitendra P. Khurana, Paramjit Khurana

**Affiliations:** Department of Plant Molecular Biology, University of Delhi South Campus, New Delhi, India; Semmelweis University, Hungary

## Abstract

Reduction in crop yield and quality due to various abiotic stresses is a worldwide phenomenon. In the present investigation, a heat shock factor (HSF) gene expressing preferentially in developing seed tissues of wheat grown under high temperatures was cloned. This newly identified heat shock factor possesses the characteristic domains of class A type plant HSFs and shows high similarity to rice OsHsfA2d, hence named as TaHsfA2d. The transcription factor activity of TaHsfA2d was confirmed through transactivation assay in yeast. Transgenic *Arabidopsis* plants overexpressing TaHsfA2d not only possess higher tolerance towards high temperature but also showed considerable tolerance to salinity and drought stresses, they also showed higher yield and biomass accumulation under constant heat stress conditions. Analysis of putative target genes of AtHSFA2 through quantitative RT-PCR showed higher and constitutive expression of several abiotic stress responsive genes in transgenic *Arabidopsis* plants over-expressing TaHsfA2d. Under stress conditions, TaHsfA2d can also functionally complement the T-DNA insertion mutants of AtHsfA2, although partially. These observations suggest that TaHsfA2d may be useful in molecular breeding of crop plants, especially wheat, to improve yield under abiotic stress conditions.

## Introduction

Plants are exposed to several environmental stresses during their life cycle that hampers their growth and development, resulting in considerable yield losses. Some of the common abiotic stresses that plants encounter are drought, flooding, salinity stress and extreme temperatures. As a consequence of a combination of different abiotic stresses, there is a dramatic yield loss in many crop plants. High temperature stress is one of the most severe stresses adversely affecting crop production worldwide [1]. However, morphological, physiological, biochemical and molecular changes occur in some plant species, that enable them to survive under high temperature conditions. A common response to high temperature stress is the synthesis of Heat Shock Proteins (HSPs). HSP encoding genes are found to be under the control of Heat Shock Factors (HSF), transcription factors that regulate the expression of HSP genes. The number of HSFs differs in organisms, for example, while vertebrates have four, *Drosophila*, *C. elegans* and yeast have only one HSF [2]–[3]. In plants, the numbers of HSFs are characteristically high, while *Arabidopsis* has 21, rice 25, *Brachypodium* 24, soybean 52 and *Poplar* 27 members [4]–[6]. Plant HSFs are represented by conserved structural features such as presence of highly conserved N-terminal, helix turn helix type DNA binding domain (DBD), followed by an oligomerization domain composed of two hydrophobic heptad repeats (HR-A and HR-B) connected to DBD by a linker of 15–80 residues and presence of nuclear localization (NLS) and nuclear export signals [4]. In contrast to N-terminal architecture, the C-terminal trans-activation domain (CTD), possessing AHA motif, is less conserved in plants. Based on sequence homology and domain architecture, plant HSFs have been divided into three conserved classes. Type A HSFs are characterized by exclusive presence of AHA transactivator motif in their C-terminal trans-activation domain (CTD), whereas class B and class C HSFs do not possess a trans-activator domain. Class B HSFs have a longer HR-A/B region due to insertion of 21 and 7 residues, respectively, and are more similar to non-plant HSFs than class A and C [4], [7]. When exposed to heat stress, these HSFs form trimers and bind with high affinity to DNA at the heat stress elements (HSEs) in the promoter sequences [8], [9]. The functional HSE sequence is inverted repeats of 5’-nGAAnnTTCn-3’ motif in promoters of the Hsp genes [10]. In plants, HSF family has been described in detail in two model organisms, *Arabidopsis* and rice [4], [5], [11]–[12].

There is functional diversification found among different HSF members. HSFA1a is described as the master regulator in tomato for acquired thermotolerance [13]. It forms a complex with other HSFs, A2 and B1, and thus regulates thermal response. Unlike tomato, *Arabidopsis* has four HsfA1 and, recently, Liu et al. [14] developed a quadruple mutant to remove functional redundancy of all *HsfA1* genes in *Arabidopsis*. It was found that more than 65% heat shock up-regulated genes were *HsfA1* dependent and, apart from severely reduced thermo tolerance, the quadruple mutant was also defective in overall growth and development, thus confirming the role of HsfA1 as the master regulator. In another recent report, triple and quadruple mutants of 4 HsfA1-type proteins were generated and it was found that HS responsiveness of *DREB2A* disappeared, indicating again *Arabidopsis* HsfA1 as the main positive regulator in HS-responsive gene expression [15]. In a genome-wide expression analysis of Hsf gene family in rice, *OsHsfA1* showed a stable expression across 28 different tissues, representing major plant developmental stages and abiotic stress conditions [5]. Unlike HsfA1, HsfA2 is expressed only in heat-stress induced plants and functions as a regulator in other environment stresses as well [16]–[17]. HsfA2 is strongly induced upon heat stress in tomato, *Arabidopsis* and rice [18]–[19], [5] and is found to play a role during heat stress or in recovery after HS [20]. It has also been reported that under oxidative stress, 26 proteosome function and/or heat shock protein (HSP90) activity inhibits, which is a trigger for the HsfA2 expression [21]. HsfA2 is also considered as an important linker between heat and oxidative stress responses [22]. AtHsfA2 knockout mutants display reduction in basal and acquired thermotolerance as well as oxidative stress while, its overexpression leads to increased tolerance under both these stressed conditions. Schramm et al. [23] functionally validated the transcription activation potential of HsfA2 on GUS reporter construct containing target promoters and their deletion analysis to verify HsfA2 binding site. This heat inducible HsfA2, when overexpressed in *Arabidopsis*, shows more tolerance to anoxia and submergence stress as well [24]. Transgenic *Arabidopsis* overexpressing OsHsfA2e showed thermotolerance in cotyledons, rosette leaves, inflorescence stems and seeds [25]. Charng et al. [26] reported direct evidence of the biological function of HsfA2 in sustaining the transcript level of HSP genes during prolonged heat stress and recovery. Further, Cohen-Peer et al. [27] reported physical interaction of AtHsfA2 and AtSUMO1, which represses its transcriptional activity. Recently, the role of OsHsfA2c and OsHsfB4b has been implicated in *OsClpB-cyt* expression [28]. Apart from heat stress, class A HSFs are also induced in *Arabidopsis* and rice by other abiotic stresses, such as cold, oxidative, salt and drought [5], [29]–[30]. In rice, *spl17* mutant defective in *OsHsfA4d*, leads to disease mimic spotted leaf phenotype under high temperature [31]. The only known HSF gene from wheat, Ta*HsfA4a* is involved in cadmium tolerance [32]. In *Arabidopsis*, over-expression of HsfA4a lead to decreased expression of cytosolic H_2_O_2_ scavenging ascorbate peroxidase and it was hypothesized that HSFs may act as H_2_O_2_ sensors in the plants [33], [34]. In plants, class A HSFs have been characterized in more detail than class B and class C HSFs. Tomato HsfB1 is a transcription co-activator, functioning along with HsfA1 and hypothesized as an HS induced factor, essential for maintenance and restoration of housekeeping gene transcription during stress conditions [35]. In *Arabidopsis,* HsfB1 is heat inducible among all five class B HSF, however, its over-expression had no effect on HSP induction and thermotolerance [19], [36]. In rice also OsHsfB1, B2a and B2b were found responsive to heat and oxidative stresses [5], [30]. Kumar et al. [37] suggested that class B HSFs act as negative regulator of biotic stress response, since down-regulation of class B Hsf resulted in increased expression of plant defense genes.

Wheat is one of the most important food crops in the world, and its complete genome has not been sequenced. Moreover, compared to rice and *Arabidopsis*, there have been few studies investigating the stress response associated genes, especially heat stress related genes in wheat, partly because of its hexaploid nature, larger genome size and relatively longer life cycle. In the present investigation, the cloning of a seed preferential heat shock factor, identified through subtractive hybridization [38] and its characterization in transgenic *Arabidopsis* plants was undertaken.

## Materials and Methods

### Plant material, growth conditions and stress treatments

Bread wheat (*Triticum aestivum* L.) cv. CPAN1676 and *Arabidopsis* ecotype Col0 were used throughout the present study. For seedling based studies, wheat plants were grown in plastic trays in a growth chamber (Conviron, Canada) and *Arabidopsis* plants were grown on half-strength MS medium in Petri plates at 20°C and 16 hour photoperiod in daily cycle. For other stages of plant development, plants were grown in potted soil and Soilrite for wheat and *Arabidopsis,* respectively. For stress treatments, plants were transferred to growth chambers set at specified temperatures, and heat stress provided for different time periods. After heat stress treatment, the control and stressed tissues were sampled, flash frozen in liquid nitrogen and stored at –80°C until RNA isolation. Remaining seedlings were transferred back to the growth chamber for recovery. For developing grains, potted wheat plants grown in departmental garden were transferred for two hours to the growth chambers maintained at 37°C and 42°C at 3,7, 14, 21 and 28 days after anthesis. Sampling of developing grains was done from spikes of control and stressed plants and developing seeds were stored at –80°C until RNA isolation. For germination assay under salt stress, *Arabidopsis* seeds were germinated on ½ MS for control condition and ½ MS supplemented with different concentrations of NaCl in Petri plates. For seedling response under salinity stress, salt stress was provided by immersing two-week-old seedlings in ½ MS medium supplemented with 150 mM and 300 mM NaCl for stress conditions. Similarly, for drought stress experiment, the seeds were germinated on ½ MS for control condition and ½ MS supplemented with 300 mM of mannitol for water stress condition.

### Cloning of wheat HSF cDNA and genomic fragments

Total RNA from developing wheat seeds was isolated as described by Singh et al. [39]. For RACE-PCR, previously identified wheat EST [38] showing homology with rice HSF was used as a template for designing the primers. Both 5`and 3`RACE-PCR was performed using SMART™ RACE amplification kit (Clontech, Palo Alto, USA) as per the manufacturer’s instructions. The resultant PCR fragments were then cloned using pGEM-T Easy vector (Promega, USA) and sequenced. For cloning of genomic fragment, primers were designed from cloned full-length cDNA and PCR was conducted by using wheat genomic DNA as template by using Long-PCR Enzyme mix (Fermentas, Lithuania). The PCR products were purified using the PCR purification kit (Qiagen, Germany) and cloned into the pGEM-T Easy vector and then sequenced.

### Sequence analysis and phylogenetic relationship

The nucleotide and protein sequences were analyzed using DNA analysis software DNASTAR. Nucleotide and the deduced amino acid sequences searched for their homology with the existing rice sequences in the NCBI database using the BlastN and BlastP programs [40]. Phylogenetic tree was constructed using neighbourhood joining method with boot strap values in CLUSTALX program by using full length protein sequences of different rice HSFs as reported earlier [5].

### Expression analysis by Northern and Semi-quantitative RT-PCR

Twenty micrograms of total RNA from control and high temperature treated samples were resolved in 1.2% agarose gel containing formaldehyde and transferred to Hybond N membrane (Amersham Pharmacia Biotech, UK). PCR-amplified cDNA fragment of EST [38] representing wheat Hsf was purified from agarose gel. Probes were labeled with α^32^P-dATP using Megaprime DNA labelling system (Amersham Pharmacia Biotech, UK) and purified through Probquant G-25 column (Amersham Pharmacia Biotech, UK). For expression analysis of wheat HSF in different developing seed tissues, a two-step RT-PCR was employed. An aliquot of 2 µg of RNA from each sample was used to synthesize the first strand cDNA using the SuperScriptIII first strand cDNA synthesis kit (Life Technologies, USA), in a 20-µL reaction volume containing 1x reaction buffer, 1 µL of oligo dT_(20)_ primer (50 µM), 2.5 mM each of dCTP, dTTP, dGTP, dATP, and 200 units of reverse transcriptase SuperscriptIII. After incubation at 50°C for 1 h, RNA was removed by incubating with RNase H at 37°C for 20 min. After RNase H treatment, 80 µL MQ water was added to each tube. 1 µL of this diluted cDNA template was added into each PCR reaction with *TaHsf* gene-specific primers (Table S1). PCR was conducted with the following program using Taq DNA polymerase (Roche, Germany): initial denaturation at 94°C for 5 min, followed with 94°C for 30 s, 60°C for 30 s, 72°C for 1 min with 25 cycles. A wheat actin was used as internal control (Table S1). The PCR products were checked on 1.2% agarose gel in 1x TAE buffer with Ethidium Bromide.

### Transactivation analysis in yeast

For the measurement of the transactivation activity, the complete *TaHsf* coding sequence fragment amplified by PCR from *pGEM-T Easy:TaHsf* plasmid was cloned between the *Nde*I and *Sma*I sites of vector pGBKT7 (Clontech, USA). The recombinant plasmids were transformed into the yeast strain AH109 harboring the *LacZ* and *HIS3* reporter genes. The *HIS3* reporter gene activity was confirmed by a viability test on a medium lacking histidine. The LacZ activity was observed in a medium containing 200 mg/L X-gal.

### Construction of binary vectors and plant transformation

For over-expression studies, a 1.39 kb fragment (cDNA) containing the *TaHsf* open reading frame along with 5’ and 3’ UTR, was PCR amplified from pGEM-T Easy:*TaHsf* plasmid with *Topo-TaHsf*-F and *TaHsf*-R primers (Table S1). The amplicon was inserted into pENTR/D-TOPO vector (Invitrogen) for construction of entry clone and sequenced. This cassette was then mobilized into the destination vector pMDC32 by LR-clonase reaction to generate pMDC32:*TaHsfA2d* vector. *Arabidopsis thaliana* Col0, wild type and T-DNA mutant plants for AtHsfA2/AT2G26150 (SALK_008978; The Salk Institute Genomic Analysis Laboratory, http://signal.salk.edu/tabout.html) were transformed by floral dip method [41]. For analysis of different transgenic lines under various abiotic stresses, T3 homozygous seeds were used and the results presented represents at least three independent experiments. The data presented represents at least 10 replicates and values are represented with average means of these experiments with standard error bars. P values were calculated by student t-test using functions in MS-Excel and represented in the bar diagrams.

### Expression analysis by RT-qPCR for HSF target genes

Total RNA from different *Arabidopsis* plants was isolated using the RNeasy plant mini kit (Qiagen, Germany) according to the manufacturer's instructions, including on-column DNaseI treatment to remove genomic DNA contamination. Twoµg of the total RNA was used as template to synthesize cDNA employing the High Capacity cDNA Archive kit (Applied Biosystems, USA) and mixed with 200 nM of each primer and SYBR Green PCR Master Mix (Applied Biosystems) for real-time PCR analysis, using the ABI Prism 7000 Sequence Detection System and Software (PE Applied Biosystems) according to the manufacturer's protocol. The specificity of the reactions was verified by melting curve analysis. At least two independent RNA isolations were used for cDNA synthesis, and each cDNA sample was subjected to real-time PCR analysis in triplicate. The primer sequences for AtHsfA2 target genes were taken from an earlier report related to characterization of AtHsfA2 target genes [16].

### Chlorophyll Fluorescence Measurements

PSII activity was measured according to Krause and Weis [42]. Measurements of modulated chlorophyll fluorescence emission from the upper surface of the leaf were made using a pulse amplitude modulation fluorometer (Junior-PAM chlorophyll fluorometer, H.Walz, Germany). Leaves were dark-adapted for 20 min before measuring the induction of fluorescence. Measurements of various PSII functions such as maximum photosynthetic efficiency (Fv/Fm), effective photosynthetic efficiency (YII) and Electrone Transport Rate (ETR) were recorded in rosette leaves at specified time points in at least ten plants per line viz. WT, mutant and transgenics.

### Measurements of total chlorophylls

Chlorophylls were estimated by the non-maceration method according to Hiscox and Israelstam [43]. Leaf samples (0.05 g) from control and salt stress treated plants in five different replicates were incubated in 5 mL of DMSO at 65°C for 4 hr in dark. Absorbance was recorded at 645, 663nm in a (Beckman DU™ 640B, Beckman Instruments Inc., USA) spectrophotometer, and chlorophyll contents were calculated according to the following formula 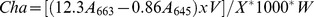


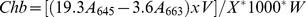



where, V =  volume of DMSO in mL, X =  path length, 1 cm,and W =  fresh weight in grams.

## Results

### Identification and cloning of seed preferential wheat Hsf

**Figure 1:**
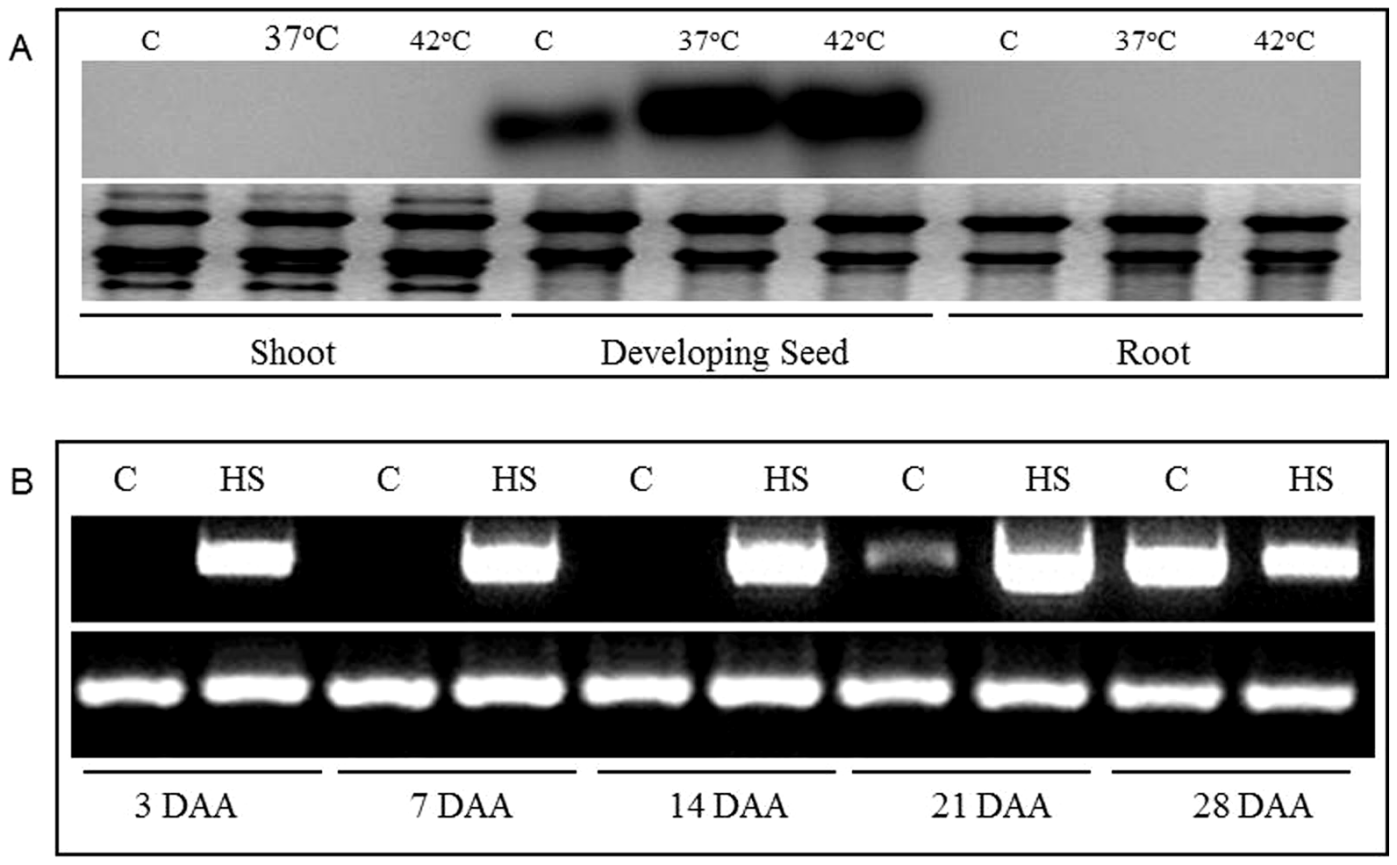
Expression analysis of wheat *TaHsfA2d.* (A) Northern blot expression analysis of EST representing wheat HSF in shoot, root tissues of two weeks old seedling and developing seed tissues. Potted plants were given heat shock at indicated temperature for two hrs and RNA from control (C) and heat shock treated (HS) plants was probed with EST as the probe (B) Expression analysis by semi quantitative RT-PCR, *TaHsf* (upper) and wheat *actin* (lower) in developing seed tissues of wheat (DAA-days after anthesis) at indicated time points. Heat stress was provided to potted plants at different time points during various stages of seed development after anthesis. The experiment was repeated two times.

Previously, we had generated a large number of wheat ESTs through subtractive hybridization of RNA extracted from control and heat stressed tissues of three different growth stages of wheat, viz. seedling, flower and developing grains [38]. One such EST showing high homology with rice Hsf, was identified from developing seed tissue. We checked the tissue specificity of this transcript by RNA gel blot analysis. Fig 1A shows that this wheat Hsf is expressed only in developing seed tissues and absent from shoot and root tissues. Although, a significant amount is also present in the control tissue, its transcript was further enhanced by heat stress at 37°C and 42°C. We then checked the inducibility of this gene at different stages of wheat grain development under control and heat stress conditions. This wheat Hsf is induced by heat stress in the early stages of seed development, and gradually become constitutive as the seed matures (Fig. 1B).

**Figure 2:**
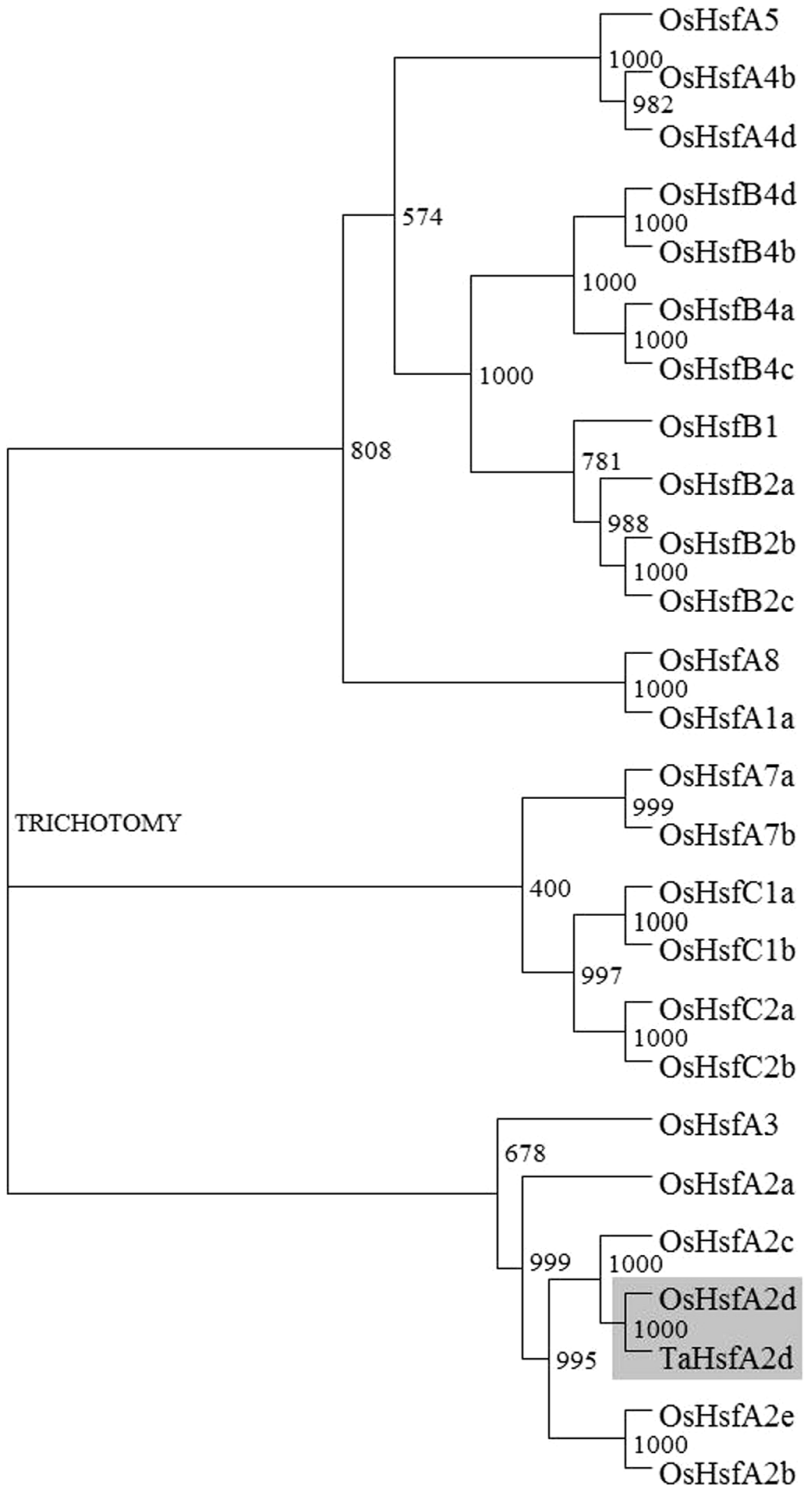
Phylogenetic relationship of TaHsfA2d with rice HSF gene family. The tree was constructed in ClustalX by neighbor joining method with boot strap values and by using deduced amino acid sequence of newly identified wheat Hsf and amino acid sequences of all known rice Hsf proteins as reported earlier in Chauhan et al. (5)

To complete the full length cDNA, 5’ and 3’ RACE-PCR (Fig. S1) was performed and a full length cDNA of 1396 bp was cloned harboring 1026 bp ORF along with 5’ UTR of 120 bp and 3’ UTR of 205 bp, along with poly-A tail. The complete nucleotide and deduced amino acid sequence was submitted to NCBI with accession number KF061193. Subsequently, a genomic clone of 2682 bp was also obtained by performing PCR using genomic DNA as template (Fig. S1). The cDNA and genomic clone alignment revealed the presence of a single intron of 1256 bp (Document S1). The deduced amino acid sequence of 341 residues was analyzed by BLAST in NCBI and TIGR databases and this transcript was confirmed as a putative HSF. To classify this wheat HSF, a phylogenetic tree was constructed by using all 25 rice HSFs. This new wheat HSF segregated as a separate clade in the resultant phylogenetic tree along with other rice class A2 type of HSFs, OsHsfA2d being the closest (Fig. 2). This was further confirmed by BLASTP analysis in NCBI and TIGR databases. TaHsfA2d shows a similarity of 69% with rice (*Oryza sativa japonica*) OsHsfA2d (Os03G06630) and a similarity of 65% with *Arabidopsis* HsfA2. With this analysis this wheat HSF was termed as TaHsfA2d.

### TaHsfA2d possesses transactivation activity

**Figure 3:**
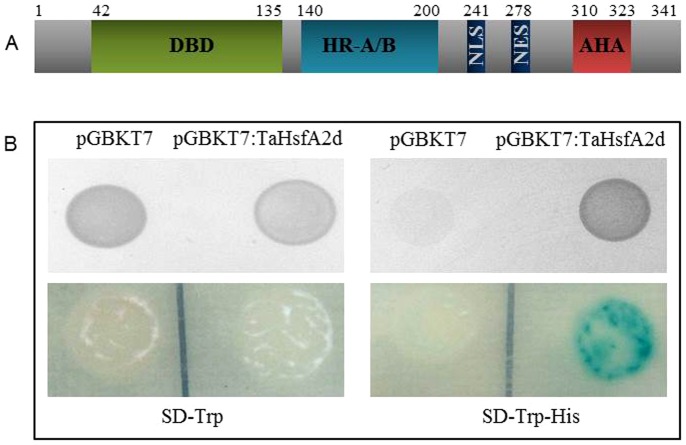
Schematic representation of wheat HsfA2d domains. (A) numbers on top represents the positions of first and last amino acid of the particular domain. Relative length and identification of different domains was done by aligning deduced amino acid sequence of TaHsfA2d with rice OsHsfA2d (Os03g06630, http://www.uniprot.org/uniprot/Q8H7Y6). (B) Transcriptional activation assay of TaHsfA2d protein in yeast (AH109). ORF of *TaHSFA2d* was fused with GAL4 DNA-binding domain in plasmid pGBKT7 and transformed in AH109 yeast strain harboring the LacZ and HIS3 reporter genes as mentioned and selected on nutrient medium lacking His and Trp (right panel). Transformed yeast cells with the pGBKT7 vector alone were used as a control (Left panel). Transcriptional activation was also checked by β-galactosidase activity of LacZ reporter gene. The experiment was repeated three times.

The amino acid alignment of TaHsfA2d with the corresponding rice homolog revealed the characteristic features of class A type HSFs, such as the presence of an N-terminal DNA binding domain, middle part containing HR-A/B, followed by potential NLS and NES and a C-terminal part containing AHA type trans-activation motif (Fig. 3A). In order to test the transactivation potential of TaHsfA2d, we used the yeast assay system. The complete ORF of TaHsfA2d was cloned in vector pGBKT7 with GAL4 DBD fusion and introduced into the yeast reporter strain AH109. The HIS3 reporter gene activity was confirmed by a viability test on histidine lacking medium, while yeast harboring pGBKT7::TaHsf2d grows on histidine lacking medium, the empty vector control showed no growth (Fig. 3B). Thus, it was hypothesized that *TaHsfA2d* has a functional AHA motif and acts as trans-activator similarly to other class A Hsfs.

### Overexpression of TaHsfA2d increases basal thermotolerance of transgenic *Arabidopsis* plants

**Figure 4:**
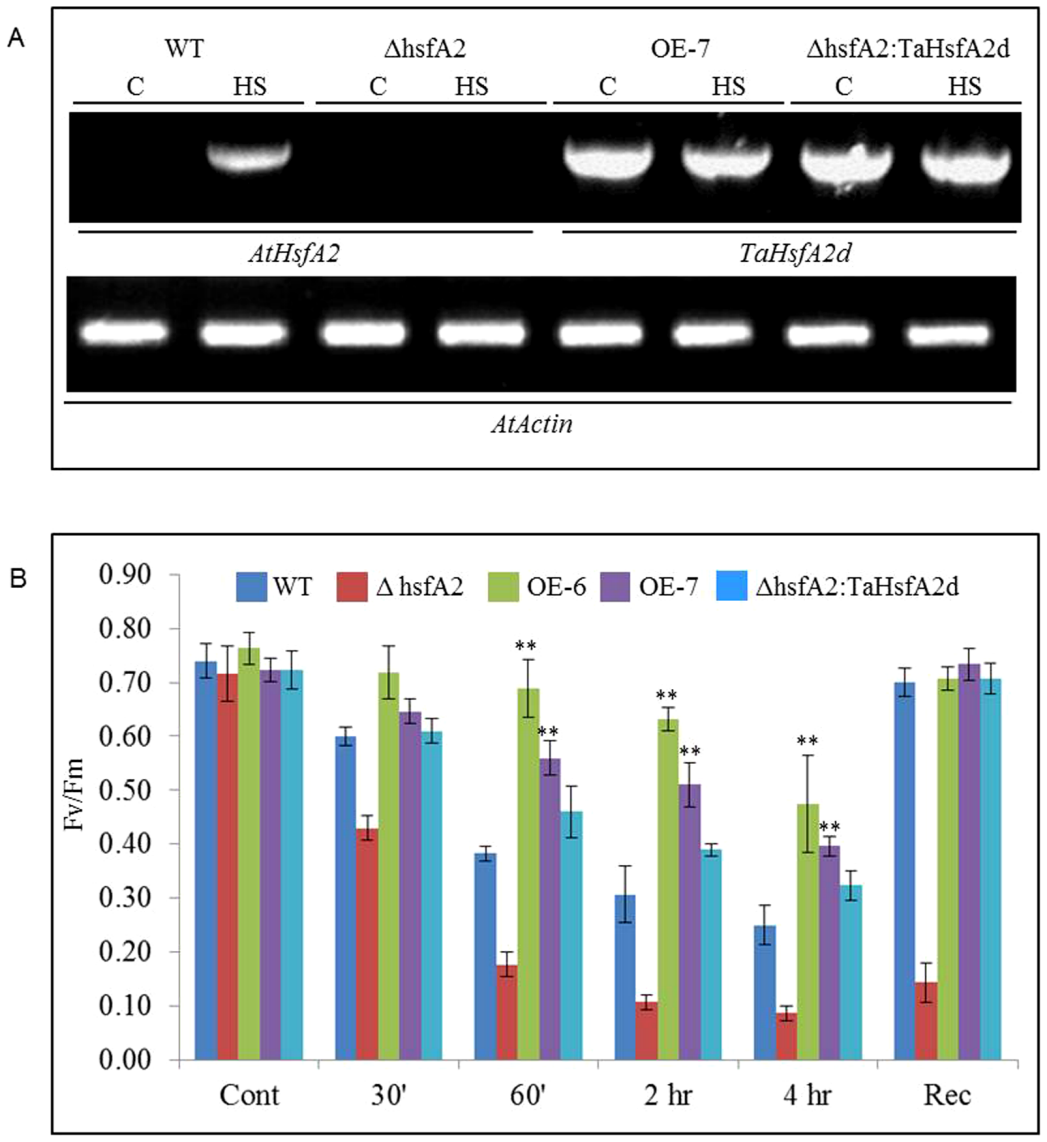
Expression analysis of *AtHsfA2* and *TaHsfA2d.* (A) Semi quantitative RT-PCR in two-weeks-old different *Arabidopsis* plants (WT, mutant and transgenics) under control and heat stress conditions, *Arabidopsis Actin* was used as internal control and the experiment was repeated three times. (B) Effect of high temperature stress of 42°C on PSII activity in terms of maximum photosynthetic efficiency (Fv/Fm) in rosette leaves of two-weeks-old different *Arabidopsis* plants (WT, mutant and transgenics). Measurements were taken by PAM fluorometer at different time points during stress and after two days of recovery. The values represent mean of at least ten individual plants of each line and the experiment was repeated three times. Error bar represents standard error (** represents p value = <0.01).

To determine the agronomical usefulness of *TaHsfA2d* in molecular breeding, the WT and mutant *hsfa2* plants of *Arabidopsis* were transformed with TaHsfA2d. The expression of native and transgene was confirmed by RT-PCR in T_2_ transgenic and WT seedlings (Fig. 4A and Figure S2). We selected and tested mutant plants for expression of AtHsfA2 after a heat shock of 30 minutes at 42°C (Fig. 4A). This was done to select the knockout (KO) *hsfa2* line (*▵hsfa2*) and it was then complemented with *TaHsfA2d* by transformation. Subsequently, the effect of a sudden heat shock of 42°C in two-week-old plants was studied by measuring the maximum photochemical efficiency (Fv/Fm) of PSII at various time points. Fig. 4B shows that sudden heat shock caused a gradual decrease in PSII activity in terms of maximum photochemical efficiency (Fv/Fm) in all the plants, however, TaHsfA2d overexpressing plants represented by two independent lines viz. OE6 and OE7 maintained a higher Fv/Fm at all the time points and showed a faster and better recovery upon returning to control temperature. In contrast, the WT and mutant plants showed drastic reduction in PSII activity right from initial phase of stress treatment, while WT plants could recover well after 2 days, mutant plants showed no measurable PSII activity.

**Figure 5:**
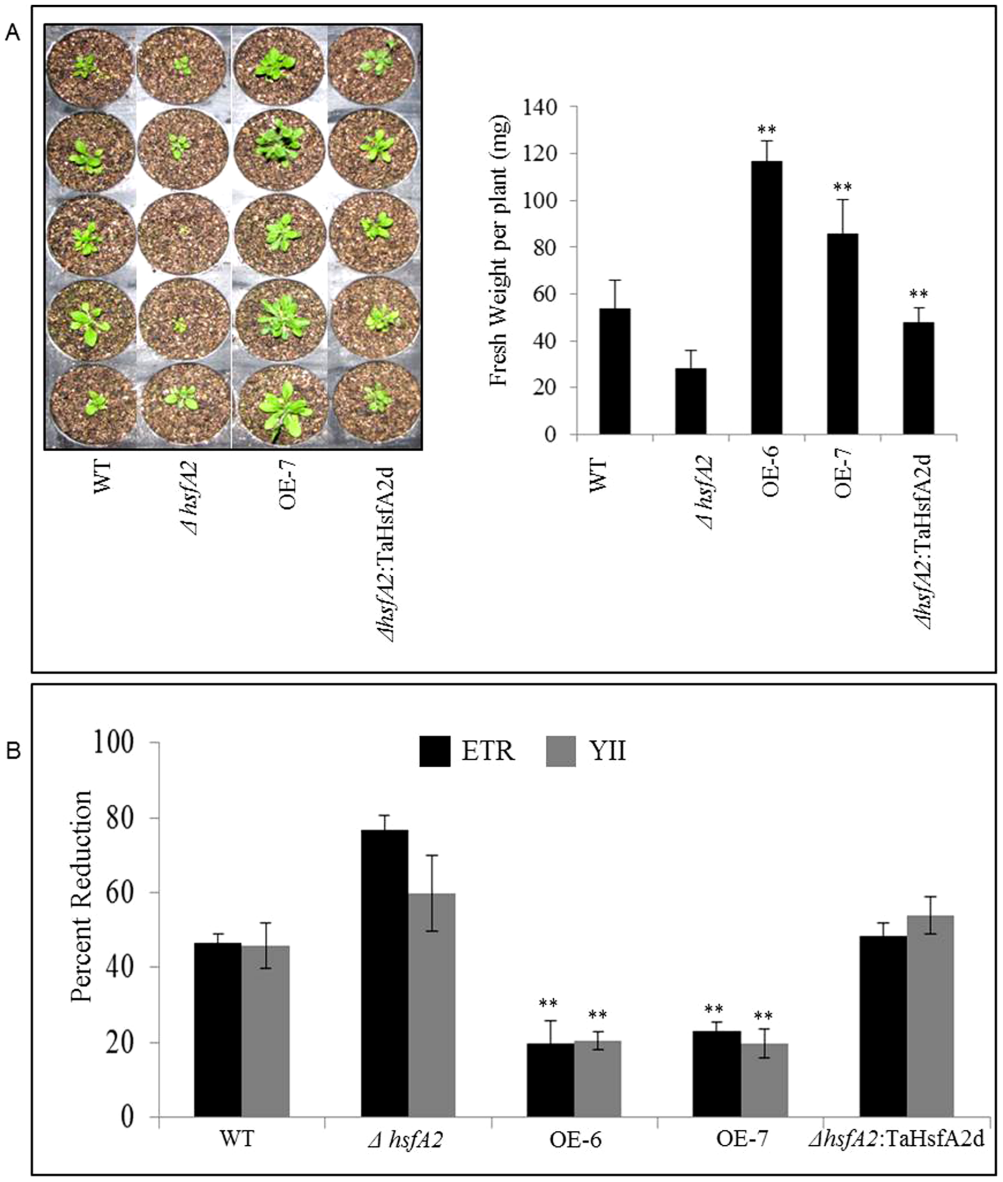
Effect of constant heat stress on growth and development of different *Arabidopsis* plants. (A) Growth and biomass accumulation in four weeks old different *Arabidopsis* plants (WT, mutant and transgenics) grown under moderate heat stress of 30°C. The seeds were first germinated at 20°C and grown for two weeks before transferring them to high temperature conditions for another two weeks and then photographed and other data was recorded. The values under fresh weight represent mean of at least ten individual plants of each line and error bar represents standard error based on three experiments (p = <0.01) (B) Steady state PSII activity in four-weeks-old *Arabidopsis* plants in terms of ETR and effective photosynthetic efficiency (YII) under continuous moderate heat stress of 30°C. The values represent mean percent reduction of at least ten individual plants of each line and the experiment was done three times and error bar represents standard error (** represents p value = <0.01).

In a separate experiment, two-week-old seedlings grown on ½ strength MS medium were transferred in small plastic tray pots in Soilrite. After establishment in soil the trays were shifted to a growth chamber maintained at moderate high temperature of 30°C for two weeks. In this moderate heat stress environment also, the overexpressing plants showed better biomass accumulation and faster growth as measured by fresh weight and number of rosette leaves (Fig. 5A). This is also evident from the steady state PSII activity measured as effective photosynthetic efficiency (YII) and ETR. Transgenic *Arabidopsis* plants overexpressing wheat TaHSFA2d showed higher PSII activity under constant heat stress conditions, showing less reduction in both YII and ETR (Fig. 5B). The mutant plants either almost ceased growth and development upon transfer to non-lethal heat stress or died, while overexpression plants had almost double the biomass than the WT plants.

**Figure 6:**
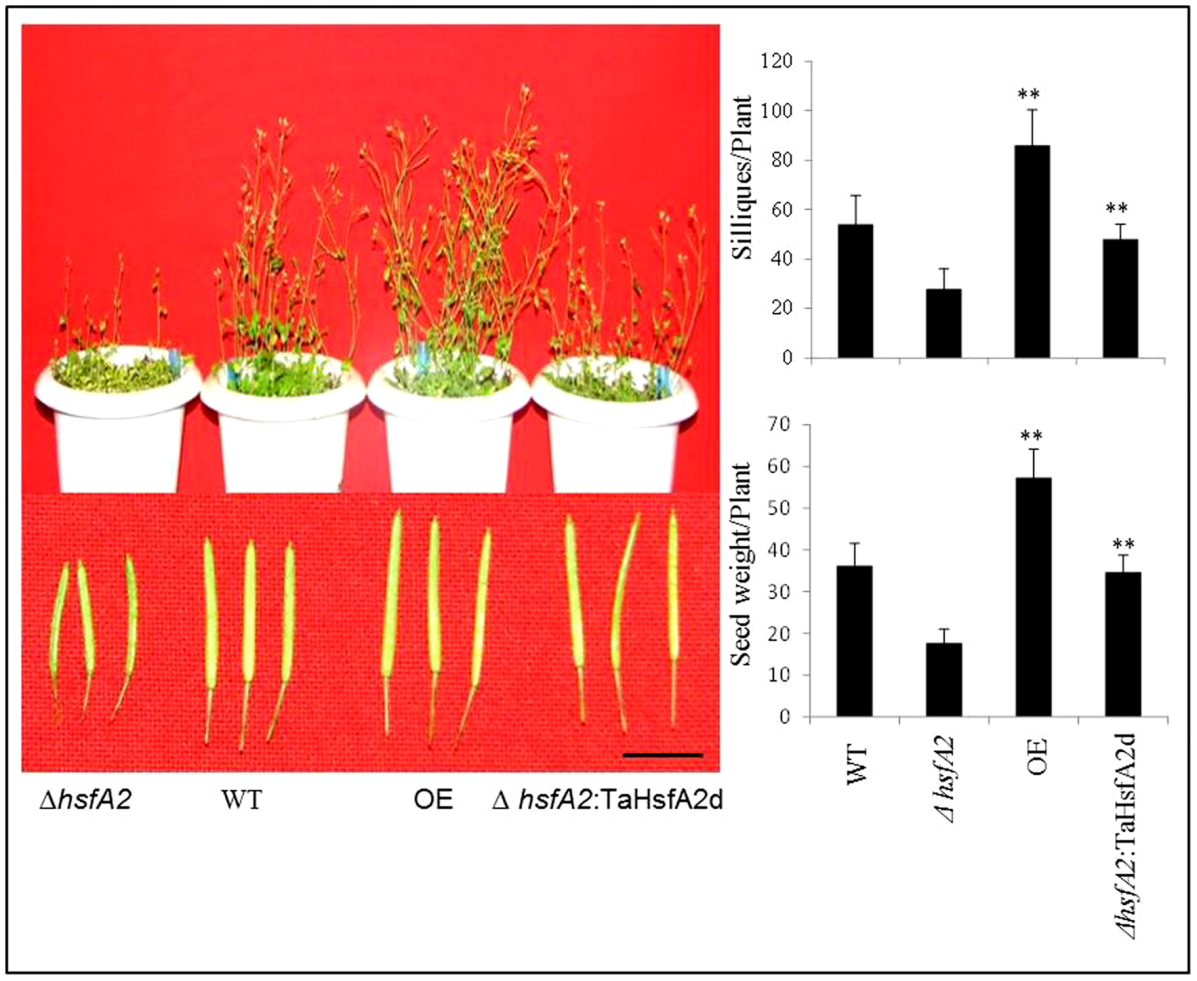
Growth and yield of *Arabidopsis* plants in terms of number of productive siliques and seed weight per plant. The plants were grown in Soilrite under constant heat stress of 30°C in a growth chamber. The data was recorded after physiological maturity and represent means of at least ten individual plants per line and error bar represents standard error (** represents p value = <0.01). The scale bar represents 1 cm.

To further check the thermotolerance of different *Arabidopsis* plants during growth and development throughout their life cycle, seeds were germinated in three inch plastic pots with 15 seeds (after germination) in each pot, and kept continuously at 30°C temperature in a growth chamber. In this assay also, the overexpression transgenic plants showed faster germination and seedling establishment. Although all the plants matured in around 8 weeks, the overexpression transgenic plants accumulated more biomass, had longer and higher number of productive siliques and showed considerably higher seed yield (Fig. 6). In comparison, the KO mutant *hsfa2* plants showed very poor growth and produced almost half the seeds as compared to WT plants and the yield was much less when compared to overexpression transgenics. This analysis showed that for most of the parameters the overexpression plants and complimented mutant plants differed significantly from WT and mutant plants, whereas WT and mutant plants behaves in a similar manner under various stresses.

### Overexpression of *TaHsfA2d* provides salt and drought tolerance to transgenic *Arabidopsis*

**Figure 7:**
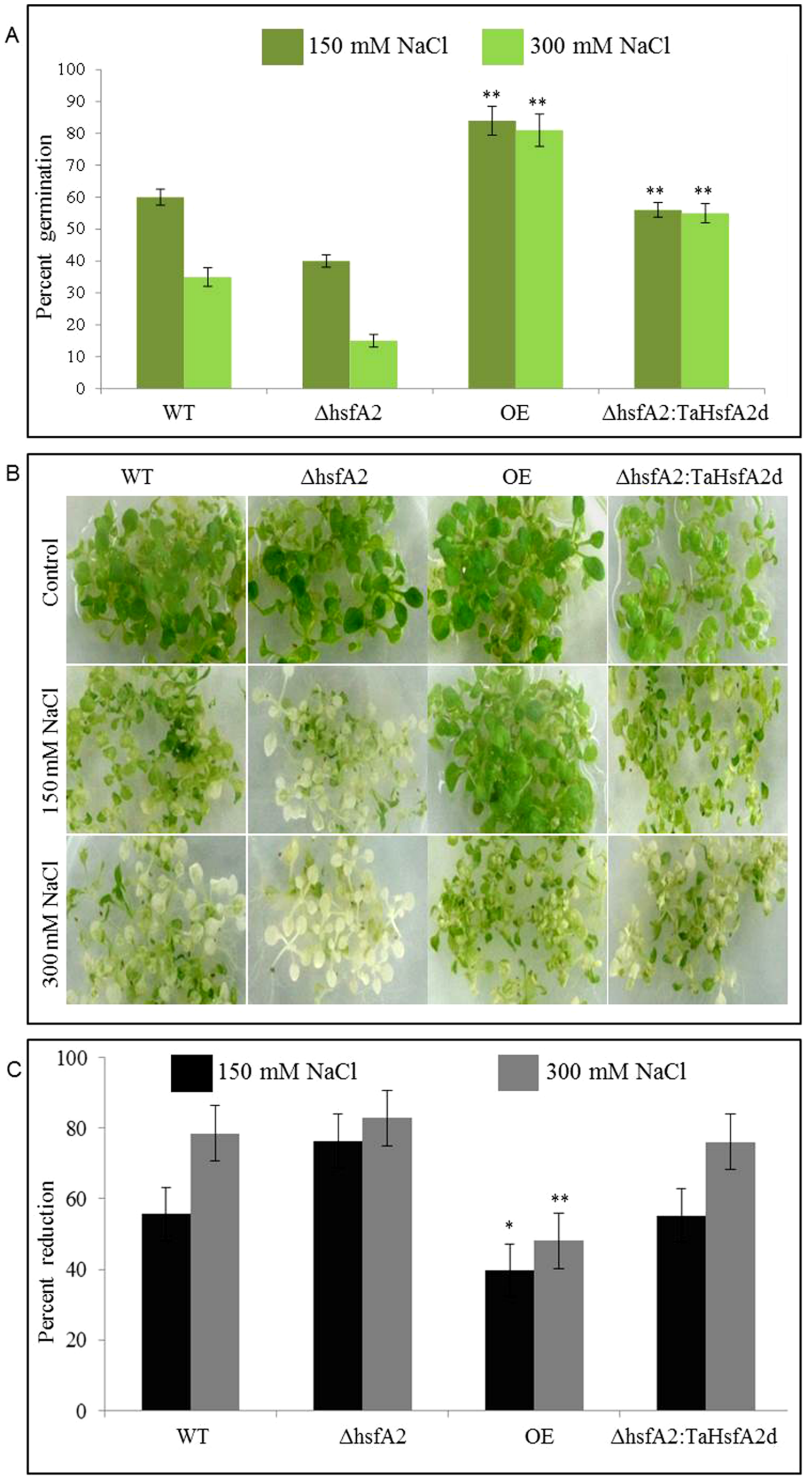
Effect of salt stress on *Arabidopsis* plants. (A) Effect of different concentrations of salt stress (NaCl) on percent germination of different *Arabidopsis* plants (WT, mutant and transgenics). *Arabidopsis* seeds were germinated on ½ MS for control condition and ½ MS supplemented with different concentrations of NaCl in Petri plates with a density of 50 seeds per plate. The data was recorded after two weeks of germination as seen in control plates and represents mean of three different plates per line. The error bar represents standard error. (B) Effect of 4 days salt stress (NaCl) during vegetative stage of two-weeks-old seedlings of different *Arabidopsis* plants. For seedling response under salinity stress, salt stress was provided by immersing two-week-old seedlings of different *Arabidopsis* plants (WT, mutant and transgenics) in ½ MS medium supplemented with 150 mM and 300 mM NaCl. (C) Effect of salt stress on total chlorophyll content. Total chlorophyll content was measured in 50 mg of plant material for each line and the data represents mean percent reduction of five replicates. The error bar represents standard error (p = 0.05(*) and 0.01(**).

**Figure 8:**
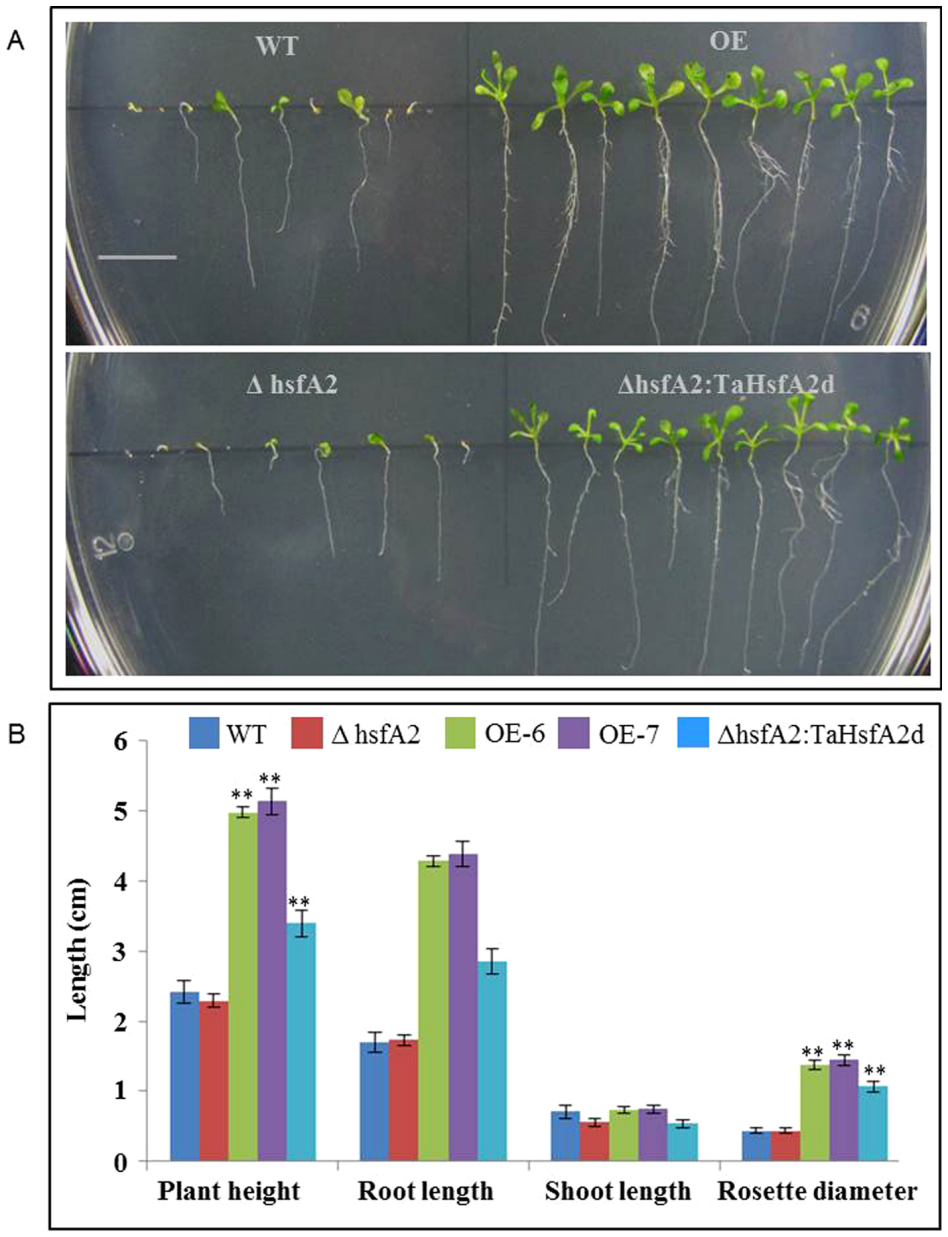
Effect of water stress on different *Arabidopsis* plants. (A) Growth and biomass accumulation of two-weeks-old different *Arabidopsis* plants (WT, mutant and transgenics) grown under drought stress caused by 300 mM mannitol (scale bar  = 1 cm). (B) Effect of simulated drought stress on some developmental parameters. The seeds were germinated on ½ MS for control condition and ½ MS supplemented with 300 mM mannitol in Petri plates. The data represents mean of ten individual plants and error bar represents standard error (** represents p = <0.01). The experiment was repeated three times.

TaHsfA2d transgenic *Arabidopsis* plants were tested for salt stress tolerance in both germinating and vegetative stages at different concentrations of NaCl. The germination potential of different *Arabidopsis* seeds viz., WT, KO, OE and KO-Complemented was checked by germinating them on ½ strength MS medium supplemented with 150 and 300 mM NaCl. OE plants showed better and faster germination of 80% as compared to 60% and 35% for WT and KO seeds respectively. The difference in per cent germination was even greater under higher salt stress condition, where OE seeds maintained the similar germination potential while only 12% of the KO plants could germinate (Fig. 7A). The salt tolerance in vegetative tissues was checked by using two-weeks-old seedlings grown on ½ strength MS media. Two-weeks-old plants of *Arabidopsis* were then dipped in ½ strength MS medium supplemented with NaCl. After four days observations were taken in terms of greening of the tissue and total chlorophyll content (Fig. 7B and 7C). The WT and KO seedlings showed a faster depletion of photosynthetic pigments than the OE plants. After 4 days exposure to salt stress, OE seedlings still maintained almost half of the original total chlorophyll content, while WT and mutant plants were almost completely devoid of chlorophyll (Fig. 7C). In a different set of experiment, we also assessed the response of different *Arabidopsis* plants towards drought stress simulated by 300 mM of mannitol. As shown in Figure 8, transgenic *Arabidopsis* plants of both the overexpression lines performed much better than both WT and mutant plants. There was a faster and robust germination in overexpressing plants and subsequently more biomass accumulation and growth was observed as measured by plant height, root-shoot length and rosette diameter (Fig. 8A and 8B).

### Constitutive expression of AtHsfA2 target genes in transgenic *Arabidopsis* overexpressing *TaHsfA2d*

**Figure 9:**
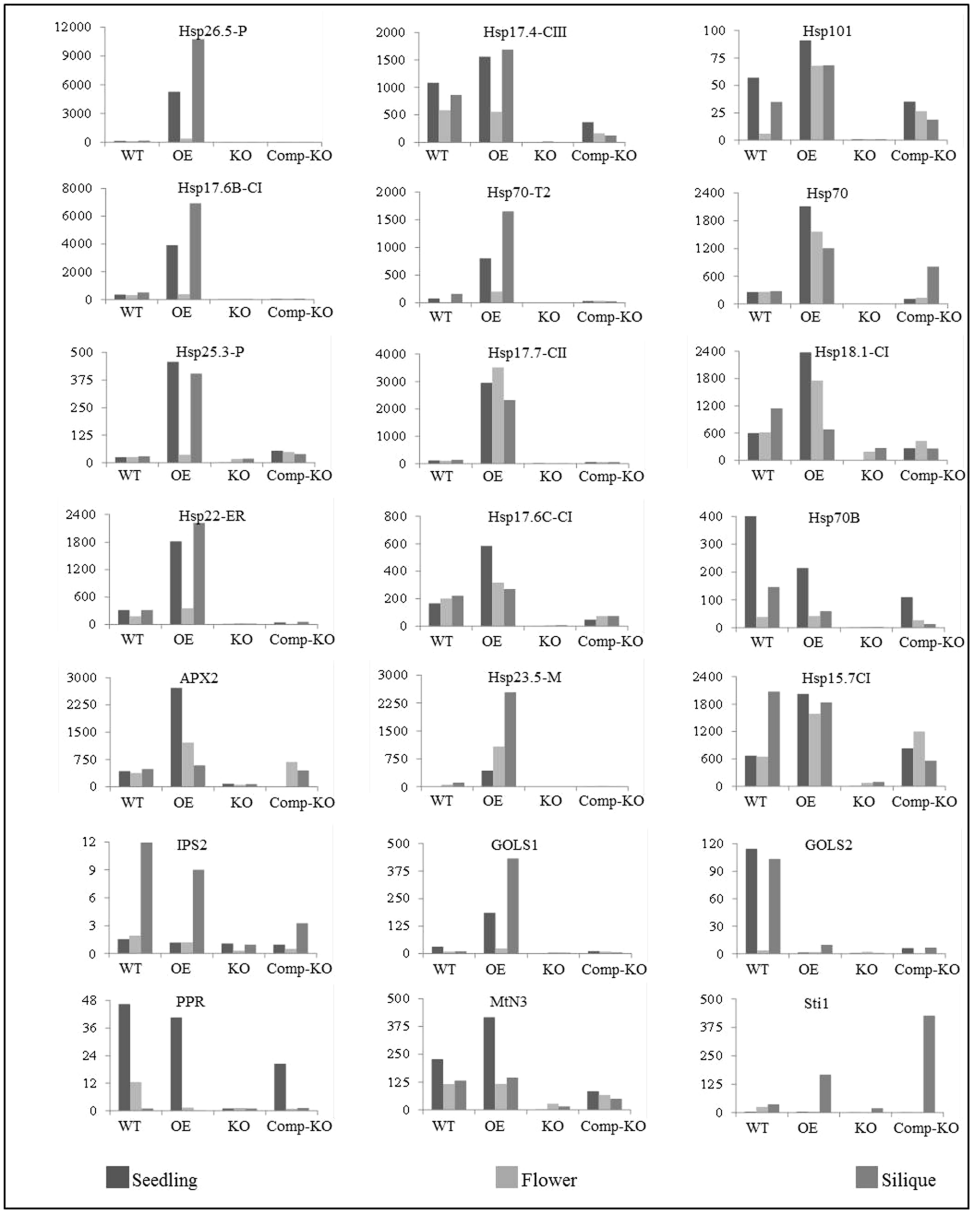
Expression analysis of some target genes of AtHSFA2. Quantitative RT-PCR was done in different tissues of *Arabidopsis* plants (two-weeks-old plants for seedling and 8-weeks-old plants for flower and siliques tissues) of WT, 35S-TaHsfA2d (OE), knock out mutant AtHsfA2 (KO) and complemented mutants with TaHsfA2d (Comp-KO). Values on Y-axis represent relative transcript abundance, and non-treated tissues were used as control ( = 1)

To understand the molecular basis of TaHsfA2d mediated stress tolerance in transgenic *Arabidopsis*, we checked the expression of AtHsfA2 target genes in WT, KO, OE and complemented KO plants by quantitative real time PCR. These genes were carefully selected on the basis of previous reports related to target genes of AtHsfA2 in *Arabidopsis* [16], [24]. For this analysis, heat stress was given at 42°C for 20 minutes only, as *Arabidopsis* class A HSFs may have functional redundancy in activating these genes. The expression of 21 target genes was examined in three different tissues, viz. seedling, flower and siliques under normal growth conditions and on exposure to heat stress conditions in these tissues (Fig. 9). These genes broadly represent different abiotic stress related genes, such as those coding for heat shock proteins (HSPs), reactive oxygen scavengers and secondary metabolites. The highest fold-change was observed for a chloroplast localized small heat shock protein (sHSP26.5); siliques showed the maximum expression, followed by seedling and flower (Fig. 9). Almost similar expression pattern was observed in case of other organelle localized sHSPs, such as ER localized sHSP22 and mitochondria localized sHSP23.5. Many of the target genes showed higher expression in siliques tissues only, such as Sti1 which is exclusively present in siliques tissue. This can be explained by the fact that TaHsfA2d was primarily identified and cloned from developing wheat seed tissues. It is noteworthy that not all the target genes have higher expression in OE plants as compared to the WT plants such as HSP70B and GolS2, suggesting that these genes may not be the primary target of TaHsfA2d in wheat. Overall, mutant plants showed little or no expression under heat stress conditions in all the three tissues in terms of relative fold change; it was also reflected by their higher Ct values when compared to WT and OE plants in the assay (data not shown).

### TaHsfA2d can partially complement *Arabidopsis* KO *hsfa2* plants

It is clearly evident from the above analysis, that *Arabidopsis* KO plants for hsfa2 are defective in heat and salt stress response. Since TaHsfA2d showed 65% homology with AtHsfA2 at the amino acid level, we tried to complement the KO plants with wheat TaHsfA2d. The complemented plants performed better than KO plants in all the experiments conducted in the present study and their response was almost at par with the WT plants (Fig. 4–8). However, we expected these plants to perform better than WT plants as the expression of the transgene, *TaHsfA2d* is governed by a strong, constitutive promoter CaMV35S. The partial recovery displayed by these complemented KO plants can be explained on the basis of relatively lower expressions of the target genes in transgenics as shown in Fig. 9. Almost all the genes showed a lower expression in terms of relative fold change than the corresponding WT and OE tissue. One exception to this is the expression of *Sti* gene in silique tissue. This discrepancy in the expression values of WT and complemented KO plants can be explained by the possible different target gene specificities of TaHsfA2d, as the wheat *TaHsfA2d* expresses preferentially in seed and its transcript levels increase substantially by high temperature treatment in seed tissues.

## Discussion

Heat shock factors have been functionally analyzed in many plants, such as *Arabidopsis*, rice, tomato and sunflower [4, 20, 25, 31, and 44]. However in wheat, only one heat shock factor, TaHsfA4d, has been functionally characterized so far [32]. In this study, we cloned and characterized a class A Hsf from wheat, TaHsfA2d, from developing wheat seeds. Through northern analysis we found that this Hsf is seed specific. Although, previous expression analysis based on EST showed that TaHsfA2d is heat inducible in both shoot and roots, nevertheless, the expression of TaHsfA2d was much higher in flower and developing seed under heat stress conditions [38]. Interestingly, in our previous study we have shown that among various class A HSFs in rice, *OsHsfA2d* also showed induced expression in developing rice seeds [5]. Apart from heat stress, we found that TaHsfA2d is also inducible by salt stress and by ABA [38]. Further, we demonstrate in this study that this TaHsfA2d functions as the transcriptional activator in yeast (Fig. 3). Unlike class B and class C Hsfs, class A Hsfs are known to be trans-activators due to the presence of AHA motif. We found that TaHsfA2d has a functional AHA motif and acts as trans-activator similarly to other class A Hsfs.

There *are* 21 Hsfs in *Arabidopsis*, 25 in rice and 24 in *Brachipodium*, thus we expect 70–75 Hsfs in wheat owing to its hexaploid nature and presence of three homeologous genomes. While both *Arabidopsis* and tomato have a single HsfA2, rice has as many as 5 different HsfA2 (a-e) [4], [12], [30], [45]. In both *Arabidopsis* and tomato, HsfA2 is highly inducible by abiotic stresses and constitutive up-regulation of this single Hsf in transgenic plants improved the tolerance to high temperature, while its down regulation showed the negative effect [13], [22], [26]. Especially in *Arabidopsis*, overexpression of AtHsfA2 increased tolerance to several types of environmental stresses, such as high temperature [19], [22], [23], [26], oxidative stress [16] and anoxia stress [24]. In rice also, different HsfA2 were found to be heat inducible and in addition, OsHsfA2d expressed in developing seeds [5]. Overexpression of OsHsfA2e increases the heat and salinity stress tolerance in *Arabidopsis* [25]. In the present investigation, *Arabidopsis* plants overexpressing TaHsfA2d showed remarkable tolerance to moderate high temperature provided continuously (Fig. 5 and 6). This could be due to the constitutive expression of several abiotic stresses related genes in the transgenic plants (Fig. 9). It has been shown earlier that overexpression of AtHsfA1a and A1b improved basal thermotolerance in transgenic *Arabidopsis* plants [46]. In the present study, a positive effect was also found on seed yield and silique length as well as silique number under moderate heat stress conditions (Fig. 6). It is known that many heat shock proteins are accumulated during seed maturation [47]. We also found higher expression of some HSPs in the siliques of transgenic *Arabidopsis* plants overexpressing TaHsfA2d (Fig. 9) such as HSP 26.5P, HSP 101, HSP70-T2, HSP 23.6-M and HSP17. Kotak et al. [48] found that accumulation of HSPs in developing seeds is regulated by AtHsfA9 in *Arabidopsis*. Whether TaHsfA2d also governs accumulation of HSPs in wheat developing grains remains to be elucidated.

Overexpression of TaHsfA2d in *Arabidopsis* also increased plant tolerance against salinity and drought stress, as reflected by better germination, higher chlorophyll retention and biomass accumulation (Fig. 7 and 8). This can again be explained by the constitutive expression of several salt/osmotic stresses related genes in transgenic plants (Fig. 8). Sun et al. [49] reported that overexpression of molecular chaperon HSP17.7CII increased salt and osmotic stress tolerance. Overexpression of GolS1 increased water stress tolerance in *Arabidopsis* through accumulation of higher amounts of raffinose [50]. In rice, it has been shown that myo-inositol 1-P-synthase (MIPS) gene has important protective functions under salinity stress [51], [52]. We have recently found that in heat stress also, the levels of myo-inositol increase significantly and which is also correlated well with *TaMIPS* expression in wheat [53]. It is therefore likely that salinity stress tolerance showed by *Arabidopsis* plants overexpressing TaHsfA2d is due to higher expression of these genes (Fig. 9).

Target genes for HsfA2 were identified through microarray analysis in transgenic plants overexpressing *Arabidopsis* HsfA2 [16] and rice HsfA2e [25]. It has been found that *Arabidopsis* HsfA2 governs expression of 46 genes, which were constitutively expressed in transgenic plants. Similarly, 37 genes were constitutively up-regulated in transgenic *Arabidopsis* plants overexpressing rice OsHsfA2e. There is a fair similarity in the diversity of genes governed by these two Hsf A2. On the basis of these reports we checked the expression of 21 target genes of HsfA2. These genes mainly represent heat shock proteins, reactive oxygen scavengers and other stress related proteins. We found that 16 out of 21 genes examined, have constitutively higher expression in seedling, flower and siliques of transgenic *Arabidopsis* plants overexpressing TaHsfA2d. A chloroplastic sHSP gene showed remarkably high expression in all the tissues tested (Fig. 9). Recently, we have shown that over-expression of a wheat chloroplastic sHSP26 improves photosynthetic efficiencies under heat stress conditions in transgenic *Arabidopsis* plants and also take part in seed development [54]. It is worth mentioning here that in our previous report [38], we reported many seed specific genes induced by heat stress, are also constitutively expressed in transgenic *Arabidopsis* plants over-expressing TaHsfA2d, such as *sHSPs, HSP70, HSP101* and *Sti*. It is therefore likely that heat, drought and salt stress tolerance of the transgenic *Arabidopsis* plants overexpressing TaHsfA2d is provided by constitutive expression of these stress related genes.

*Arabidopsis* plants overexpressing TaHsfA2d, which preferentially expresses in developing wheat grains, showed improved heat and salt stress tolerance at various growth stages, and higher yields under moderate heat stress conditions. These findings suggest that TaHsfA2d gene may be useful in molecular breeding of wheat for enhanced thermotolerance especially during terminal heat stress.

## Supporting Information

Figure S1 **Cloning of wheat Hsf cDNA and Genomic clones.** 5` and 3` RACE-PCR was done by using RNA from developing seeds from the heat stressed plants. Both 5`and 3`RACE-PCR was performed using SMART™ RACE amplification kit (Clontech, Palo Alto, USA) as per the manufacturer’s instructions. The resultant PCR fragments were then cloned using pGEM-T Easy vector (Promega, USA) and sequenced. For cloning of genomic fragment, primers were designed from cloned full-length cDNA and PCR was conducted by using wheat genomic DNA as template by using Long-PCR Enzyme mix (Fermentas, Lithuania). The PCR products were purified using the PCR purification kit (Qiagen, Germany) and cloned into the pGEM-T Easy vector and then sequenced.
(TIF)

Figure S2 **Confirmation of stable transgenic** ***Arabidopsis*** **lines by expression analysis of** ***TaHsfA2d*** **in different transgenic lines by RT-PCR.** First strand cDNA was made by using Superscript III first strand cDNA synthesis system (Life Technologies, USA) and RNA from leaf samples of WT plants and plants from different OE *TaHsfA2d Arabidopsis* lines and then PCR was done with *TaHsfA2d* specific primers.
(TIF)

Table S1 List of primers.
(DOC)

Document S1 **Nucleotide and amino acid sequences of TaHsfA2d.**
(DOC)
