# Supplementary figures and images for: A Seed Preferential Heat Shock Transcription Factor from Wheat Provides Abiotic Stress Tolerance and Yield Enhancement in Transgenic Arabidopsis under Heat Stress Environment
Source: PLoS One. 2013 Nov 12;8(11):e79577. doi: 10.1371/journal.pone.0079577 (PMC3827158; doi:10.1371/journal.pone.0079577)

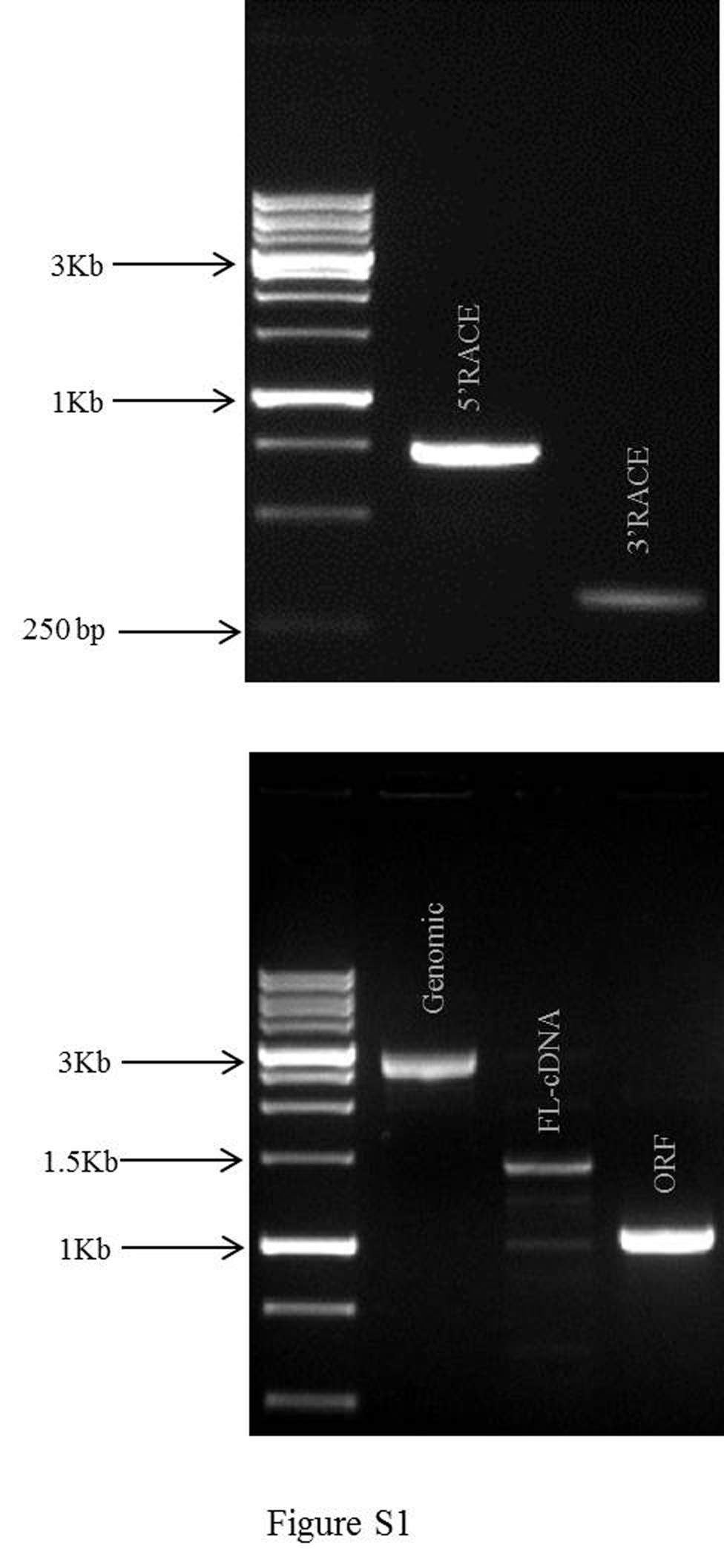

Supplement: Figure S1 — Cloning of wheat Hsf cDNA and Genomic clones. 5` and 3` RACE-PCR was done by using RNA from developing seeds from the heat stressed plants. Both 5`and 3`RACE-PCR was performed using SMART™ RACE amplification kit (Clontech, Palo Alto, USA) as per the manufacturer’s instructions. The resultant PCR fragments were then cloned using pGEM-T Easy vector (Promega, USA) and sequenced. For cloning of genomic fragment, primers were designed from cloned full-length cDNA and PCR was conducted by using wheat genomic DNA as template by using Long-PCR Enzyme mix (Fermentas, Lithuania). The PCR products were purified using the PCR purification kit (Qiagen, Germany) and cloned into the pGEM-T Easy vector and then sequenced. (TIF) [file pone.0079577.s001.tif]

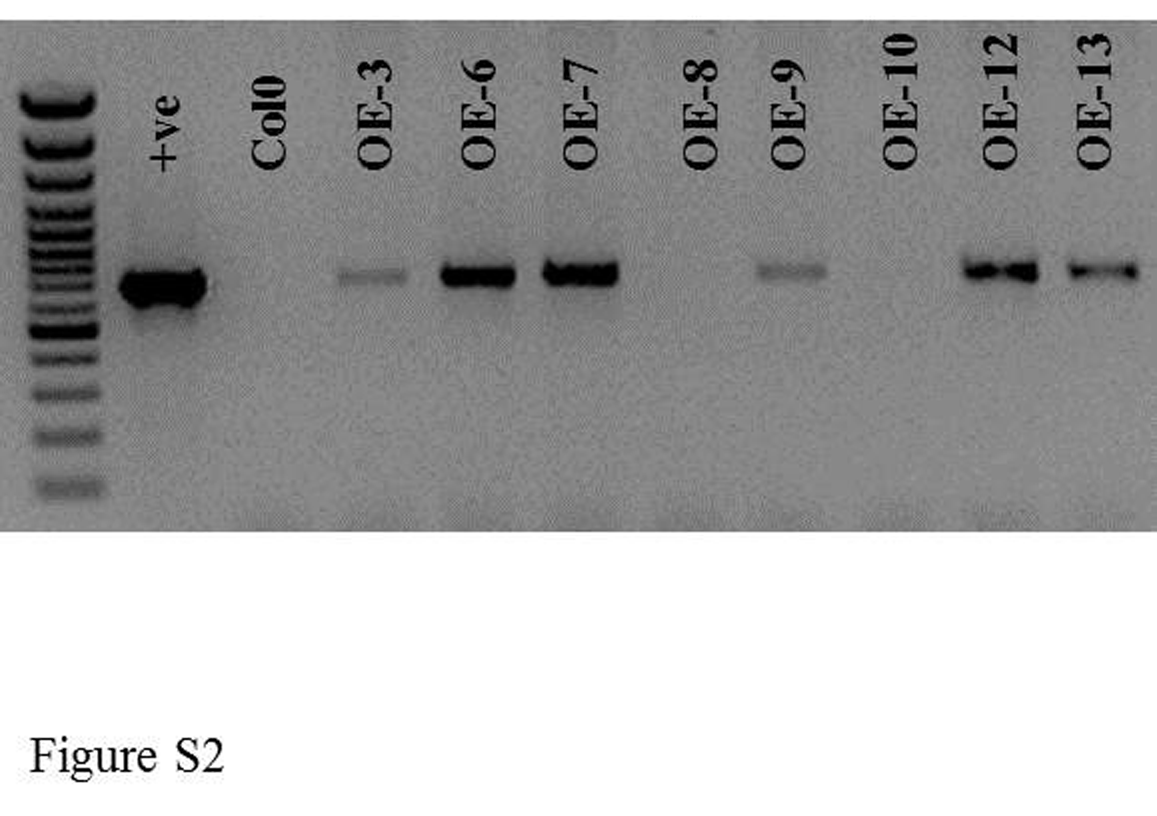

Supplement: Figure S2 — Confirmation of stable transgenic Arabidopsis lines by expression analysis of TaHsfA2d in different transgenic lines by RT-PCR. First strand cDNA was made by using Superscript III first strand cDNA synthesis system (Life Technologies, USA) and RNA from leaf samples of WT plants and plants from different OE TaHsfA2d Arabidopsis lines and then PCR was done with TaHsfA2d specific primers. (TIF) [file pone.0079577.s002.tif]
