# Supplementary material for: A Seed Preferential Heat Shock Transcription Factor from Wheat Provides Abiotic Stress Tolerance and Yield Enhancement in Transgenic Arabidopsis under Heat Stress Environment
Source: PLoS One. 2013 Nov 12;8(11):e79577. doi: 10.1371/journal.pone.0079577 (PMC3827158; doi:10.1371/journal.pone.0079577)
Supplement: Table S1 — List of primers. (DOC) [file pone.0079577.s003.doc]

| **Gene** | **Primer ID** | **Sequence (5’-3’)** |
| --- | --- | --- |
| *TaHSF* | *Topo-TaHsf*-F | caccgagccagccaaagaccacctgcg |
|  | *Topo-TaHsf*-R | gccgatgagaatccagggatagtttttatacgac |
| *AtHSFA2* | AtHsf-F | ATGGAAGAACTGAAAGTGGAAATGG |
|  | AtHsf-R | TTAAGGTTCCGAACCAAGAAAACCC |
| *Ta Actin* | Forward | GATACACGCTTCCTCATGCTATCC |
|  | Reverse | AGAGCCACCGATCCAGACACTG |

Supplementary Table S1: List of primers used for semi-quantitative RT-PCR
