# Supplementary material for: A Seed Preferential Heat Shock Transcription Factor from Wheat Provides Abiotic Stress Tolerance and Yield Enhancement in Transgenic Arabidopsis under Heat Stress Environment
Source: PLoS One. 2013 Nov 12;8(11):e79577. doi: 10.1371/journal.pone.0079577 (PMC3827158; doi:10.1371/journal.pone.0079577)
Supplement: Document S1 — Nucleotide and amino acid sequences of TaHsfA2d. (DOC) [file pone.0079577.s004.doc]

**TaHsfA2d-Nucleotide sequence of genomic clone (sequence in lower case represents intron):**

GAGCCAGCCAAAGACCACCTGCGTTGCGTGCCACTGTGCTACCCAATTCTTTGGTTCTTTGGATTCTTCCGTTTGCGACAGGAGATCGCTGGTTGTTTGCTTGCTCGGCCACGAGGAGGAATGGACCGGGTGCTGCTGCCGGTGAGGGTGAAGGAGGAGTGGCCGCCGCCGCCGCCGGAGGAGGAGGAGGAGTTGGAGCACGGGGGCCTGGCGCCGCGGCCGATGGAGGGGCTGCACGAGACTGGCCCGCCGCCGTTCCTGACCAAGACGTTCGACCTGGTGGCGGACCCGGCCACCGACGGCGTCGTCTCCTGGGGCCGCGCCGGGAACAGCTTCGTCGTCTGGGACCCGCACCTCTTCGCCGCCGTGCTGCTCCCGCGCTTCTTCAAGCACAGCAACTTCTCCAGCTTCGTCCGTCAGCTCAACACCTACGtgagtatctcctctcctggcctgcctcctcctctggttgcctagtaaggttactcagtcattacttgttcagttaacctccacttgcgagacgcttgcttgtgttgttgcaattacaatcttgcatttgtagaaccaagattttagccgagtcgctcgagcaagagatgacccaaaaatgatatatatcagggcaacatttggtgctcgtcttacaactgtggtgactatggtggtcaccatgaatattggtatgcatctgattctggaatgacataaacatacaaaaacaaatatataagatggttcagtttggtagttgtgtacgaattatgaaagaagagtaacatttatagcttctcttagcatctagcggtaagcggttctatatggcagtgaactttactagttgccggcatgttttttgtgtgtgttggtgttggctgtgtgcatccagaggccgggtgtatactcattgtgttttgtatccgcttgatgcttcattttgaactaataaaatccaccctttgtcgaaaaaattcatggcagtgaactatgttactgtgattctcttccctttgtgtaaatcttgtatatctacaattgatcagcaaagaagctgcaaactagaattatttgttttttgcctctggtttgaagggtgaacatagcttgatcttcaacagactacgatagctatggtactagtactcctactaggatagataggatccttcaatatctctgccactatctgcttacgtaaagtcatggagaatcattggattcgatggaaaaatgaggttggggccaatcccttggcagaggcatgtgcatagaaccttatttcctgaattcttcttgatacttgagaacaaggagatgctgggaagctgaatagcctgcctcttcttgacgaacatatttaatttctgacatggtctttctccaacttcttactgtgttgctagatggatcaagacaccggctgaagctacaagaacctgcttatttgttctagaagactcctttcatcaccatcaagcaatatagtatactactacctattcttccactacttataaatttgggcataacactatgtgaaacctaccattttatatgttgtgaagtatgccatgctagttgagctttgctgcttgtggcttctgaacttgagctaactgcagttgttctgctactctttgtgtttctcgaaactaaaataaatatgtgacttgttctgcaatttgcaggGTTTTAGAAAGATCGATCCAGACAGATGGGAATTCGCGAACGAGGGTTTCATTCGGGGCCAGAGACAGCTTCTAAAGATGATAAAGAGGAGGAGACCATTGTCCTATCTCCCTTCACCACAACATCAGGCCCTTGGCTCCTGCCTCGAGGTTGGCCAGTTCGGATTGGACGAAGAAATCGAAGTGCTAAAGCGCGACAAGAACGCCTTACTCGAAGAGGTGGTGAAACTGAGGCAGGAGCAGCAGAGCAGCGGAGCTGACATGCGAGCCATGGAAGAGAGGCTGCACCGCGTCGAGCAGAAGCAGCTCCAGATGATGGGTTTCCTGGCAAGAGCAATACAGAACCTTGACTTCTTTCTTCAGTTGATCCAGCAACAAGATAAACTGAAGGATCTTGAGGACGCTTATTCGAACAAGAGGAGGAGGTCCATCGACGTAATGCCGTTCCTTGGCCCTGAGGGGGCCAGTCAGAGTGAGCCACTCGAGTCCACATTCATATTTGAGGACAGGGAATTTTCAGAGCTGGAGAATTTAGCCATGAACATTCAGGGGATCAGGAAGGGCATGGAGGGTGACAGAGGTGGTCGGGATCAAGGCTGCGGTGAGGCCGAACTGACTGACGACTTCTGGGAGGAGCTGCTGAGTGAAGGAATGAGGGATGAAGCTGAGATGGTAGAGCTGGAAAGGAGGAGATCTAGATATGTCGACGCGTAGCGCAAAGTATGGGTCATTTAAGTAACAGCGATCCAAAATCTACCAGAATTCATAACCATTTAATCTATATACCATTGTATTGATAGCTGTATTGTATATTGTAGTGCTAATTCATAGTCATAGACTGATTGTATTGCGGTTCCACACCGATTATGTGGTAGTATATATCTAGTCGTATAAAAACTATCCCTGGATTCTCATCGGCAGTTTTCTACTCTGATGATCTTTGTTCATACTCAGGCCTACTGAATGAATGATTGTTCTTGGTTTAGAATCTGCC

**TaHsfA2d-Nucleotide sequence of cDNA clone accession number KF061193 (sequence in lower case represents 5` and 3`UTR with poly A-tail respectively):**

gagccagccaaagaccacctgcgttgcgtgccactgtgctacccaattctttggttctttggattcttccgtttgcgacaggagatcgctggttgtttgcttgctcggccacgaggaggaATGGACCGGGTGCTGCTGCCGGTGAGGGTGAAGGAGGAGTGGCCGCCGCCGCCGCCGGAGGAGGAGGAGGAGTTGGAGCACGGGGGCCTGGCGCCGCGGCCGATGGAGGGGCTGCACGAGACTGGCCCGCCGCCGTTCCTGACCAAGACGTTCGACCTGGTGGCGGACCCGGCCACCGACGGCGTCGTCTCCTGGGGCCGCGCCGGGAACAGCTTCGTCGTCTGGGACCCGCACCTCTTCGCCGCCGTGCTGCTCCCGCGCTTCTTCAAGCACAGCAACTTCTCCAGCTTCGTCCGTCAGCTCAACACCTACGGTTTTAGAAAGATCGATCCAGACAGATGGGAATTCGCGAACGAGGGTTTCATTCGGGGCCAGAGACAGCTTCTAAAGATGATAAAGAGAAGGAGACCATTGTCCTATCTCCCTTCACCACAACATCAGGCCCTTGGCTCCTGCCTCGAGGTTGGCCAGCTCGGATTGGACGAAGAAATCGAAGTGCTAAAGCGCGACAAGAACGCCTTACTCGAAGAGGTGGTGAAACTGAGGCAGGAGCAGCAGAGCAGCAGAGCTGACATGCGAGCCATGGAAGAGAGGCTGCACCGCGTCGAGCAGAAGCAGCTCCAGATGATGGGTTTCCTGGCAAGAGCAATACAGAACCCTGACTTCTTTCTTCAGTTGATCCAGCAACAAGATAAACTGAAGGATCTTGAGGACGCTTATTCGGACAAGAGGAGGAGGTCCATCGACGTAATGCCGTTCCTTGGCCCTGAGGGGGCCAGTCAGAGTGAGCCACTCGAGTCCACATTCATATTTGAGGACAGGGAATTTTCAGAGCTGGAGAATTTAGCCATGAACATTCAGGGGATCAGGAAGGGCATGGAGGGTGACAGAGGTGGTCGGGATCAAGGTTGCGGTGAGGCCGAACTGACTGACGACTTCTGGGAGGAGCTGCTGAGTGAAGGAATGAGGGATGAAGCTGAGATGGTAGAGCTGGAAAGGAGGAGATCTAGATATGTCGACGCGTAGcgcaaagtatgggccatttaagtagcagcgatccaaaatctaccagaattcataaccatttaatctatataccattgtattgatagctgtattgtatattgtagtgctaattcatagtcatagactgattgtattgcggttccacaccgattatgtggtagtatatatctagtcgtataaaaactatccctggattctcatcggcaaaaaaaaaaaaaaaaaaaaaaaaaaaaaaaaaaaaaaaaaaaa

**TaHsfA2d-deduced amino acid sequence (341 residues):**

MDRVLLPVRVKEEWPPPPPEEEEELEHGGLAPRPMEGLHETGPPPFLTKTFDLVADPATDGVVSWGRAGNSFVVWDPHLFAAVLLPRFFKHSNFSSFVRQLNTYGFRKIDPDRWEFANEGFIRGQRQLLKMIKRRRPLSYLPSPQHQALGSCLEVGQLGLDEEIEVLKRDKNALLEEVVKLRQEQQSSRADMRAMEERLHRVEQKQLQMMGFLARAIQNPDFFLQLIQQQDKLKDLEDAYSDKRRRSIDVMPFLGPEGASQSEPLESTFIFEDREFSELENLAMNIQGIRKGMEGDRGGRDQGCGEAELTDDFWEELLSEGMRDEAEMVELERRRSRYVDA
